# Cognitive impairment and symptoms of depression among geriatric patients in a tertiary care unit in Sri Lanka

**DOI:** 10.4103/0019-5545.71000

**Published:** 2010

**Authors:** Chaturaka Rodrigo, Sanja Perera, Madura Adhikari, Anoja Rajapakse, Senaka Rajapakse

**Affiliations:** Department(s) and Institution(s): University Medical Unit, National Hospital of Sri Lanka, UK. E-mail: chaturaka.rodrigo@gmail.com; 1 Department of Clinical Medicine, Faculty of Medicine, University of Colombo, UK; 2 Department of Clinical Medicine, Newark Hospital, Sherwood Forest NHS trust, Newark, UK

Sir,

The only study to assess prevalence of dementia in elderly in Sri Lanka has reported a prevalence of 3.98% in a semi urban population.[1] There is no data on prevalence of cognitive impairment and depression for hospitalized elderly patients, which is a major shortcoming in geriatric care.

We conducted a preliminary study to assess cognitive impairment and depression in a sample of geriatric patients presenting to the University Medical Unit of the National Hospital of Sri Lanka (the premier tertiary care hospital of Sri Lanka). This prospective study included all patients over 65 years, who were admitted over four weeks. The Mini Mental State Examination (MMSE, 30 items) and the geriatric depression scale (GDS) were used to screen for cognitive impairment and depression respectively.

We enrolled 100 eligible patients to the study [mean age: 71.1 years (standard deviation–6.324), (male 51%, female 49%)]. The percentages of gender and ethnicity did not differ significantly from the population average (*P*>0.05).[2] On GDS, Thirty one patients (31%) scored less than 5 (depression unlikely) while 34 (34%) scored within 6-10. Thirty five (35%) had scores ≥11 (depression very likely) (mean: 7.81, SD - 4.22). In assessing cognitive impairment, adjustments were made to the scoring of MMSE considering the norms for Sri Lankan elderly and level of education.[3] There were 24 (57.1%) people out of 42 who had not received a secondary education scoring less than 23 (sensitivity 86%, specificity 73%). Thirty two (55.2%) from 58 people who had a secondary education scored at or below 23 (sensitivity 71%, specificity 100%).

We also assessed the impact of educational level and social support on cognitive impairment with a statistical model. It was hypothesized that better education and family support will protect against cognitive impairment. However (holding age and gender constant), the cognitive impairment did not correlate with either factor, alone or in combination (UNIANOVA, *P*>0.05).

These observations raise two issues;

- The percentage of depressive symptoms (>60%) and cognitive impairment (>50%) was very high
- The assumption that better education and family support leads to less cognitive impairment could not be validated

The symptoms of depression may be due to illness itself. Yet it underscores the lack of psychiatric input in medical wards as none of these patients were referred to a psychiatrist. The second observation may be due to:

- Errors in variables that defined family support (frequency of visits by relations and living with family), which may not indicate coherent family functioning
- Pathology of dementia
- Depressive pseudo dementia
- Sampling errors

The findings of this preliminary study are significant and indicate the

- Importance of improving mental health among geriatric patients
- Need for coordinating psychiatric and medical care to reduce disease burden
- Need for routine screening of elderly patients for cognitive impairment
- Need for a large multi center hospital-based study to assess the interaction of socioeconomic factors, depression, dementia and disease burden
